# Dynamics of cancerous tissue correlates with invasiveness

**DOI:** 10.1038/srep43800

**Published:** 2017-03-06

**Authors:** Ann-Katrine Vransø West, Lena Wullkopf, Amalie Christensen, Natascha Leijnse, Jens Magelund Tarp, Joachim Mathiesen, Janine Terra Erler, Lene Broeng Oddershede

**Affiliations:** 1 Niels Bohr Institute, University of Copenhagen, Blegdamsvej 17, 2100 Copenhagen, Denmark; 2 Biotech Research & Innovation Centre (BRIC), University of Copenhagen, Ole Maaløes Vej 5, 2200 Copenhagen, Denmark

## Abstract

Two of the classical hallmarks of cancer are uncontrolled cell division and tissue invasion, which turn the disease into a systemic, life-threatening condition. Although both processes are studied, a clear correlation between cell division and motility of cancer cells has not been described previously. Here, we experimentally characterize the dynamics of invasive and non-invasive breast cancer tissues using human and murine model systems. The intrinsic tissue velocities, as well as the divergence and vorticity around a dividing cell correlate strongly with the invasive potential of the tissue, thus showing a distinct correlation between tissue dynamics and aggressiveness. We formulate a model which treats the tissue as a visco-elastic continuum. This model provides a valid reproduction of the cancerous tissue dynamics, thus, biological signaling is not needed to explain the observed tissue dynamics. The model returns the characteristic force exerted by an invading cell and reveals a strong correlation between force and invasiveness of breast cancer cells, thus pinpointing the importance of mechanics for cancer invasion.

Cancer is initiated by an uncontrolled cell division, but as long as the inappropriately dividing cells respect the basal membrane as the tissue border, the disease is called non-invasive or benign, and the disease can be treated by surgery. The actual life-threatening systemic disease requires another cellular quality, the ability to infiltrate into healthy tissue and spread to distant organs. Cancer cells can migrate by different modalities; besides the classical single cell migration, collective movements of cell groups and sheets have been observed^1^,^2^,^3^. Most cancerous tissues are carcinomas, which originate from epithelial cells^4^. Epithelial tissues are characterized by strong intercellular interactions, mainly provided by tight junctions, which not only guarantee mechanical support and protection, but also support collective cell behavior. One example is the cooperative cell motility during the closure of wounds. Here, epithelial cells are found to migrate in a collective fashion with long range velocity fields and definable leader cells^5^. Long-range correlation in tissue dynamics has also been observed in endothelial tissue, where well-ordered vortex patterns emerge several cell diameters away from the cell division site^6^. Individual cells need to exert a force in order to initiate tissue migration and it has been shown that local cellular migration follows the local maximum stress^7^, however, with a robust cellular collective drive to fill unfilled space^8^. Mechanical waves guiding such motion have been shown to build up in epithelial monolayers^9^. In collective migration of cancerous tissue the cells are connected via cell-cell junctions, and invasion is initiated and maintained by signaling pathways that control cytoskeletal dynamics and turnover of cell-matrix and cell-cell junctions^10^. However, it has proven difficult to define the rate-limiting mechanisms governing invasive migration, and cancer cell invasion is currently regarded as a heterogeneous and adaptive process^10^. During invasion cancer cells are subject to considerable forces that have been shown to be large enough to cause nuclear envelope rupture and DNA damage as the cells squeeze through tight interstitial spaces^11^.

Here, we take an alternative view on cancer tissue dynamics with the goal of understanding which of the observed properties can be understood alone from a materials science point of view, without the need to invoke complex signaling mechanisms, although many such signaling pathways have been identified^12^. We characterize the dynamics of cancer tissue of different invasive potential, originating from both mouse and human. As uncontrolled cell division is a hallmark of cancerous tissue, we focus on the dynamics related to cell division and on the forces exerted by the dividing cells on the surrounding tissue. We find a strong correlation between the velocity, divergence and vorticity fields of the cancer and its invasive potential. To understand the dynamics from a mechanical point of view, we formulated a model which considers the tissue as a viscoelastic continuum and reproduces well the velocity field. The model allows for quantification of the force exerted by the dividing cells on the surrounding tissue, and this force is found to correlate with the invasiveness of the cancer. These results are useful for understanding the underlying fundamental mechanisms of cancer tissue dynamics.

## Results

### Characterizing the dynamics of cancerous tissue

**Figure 1:**
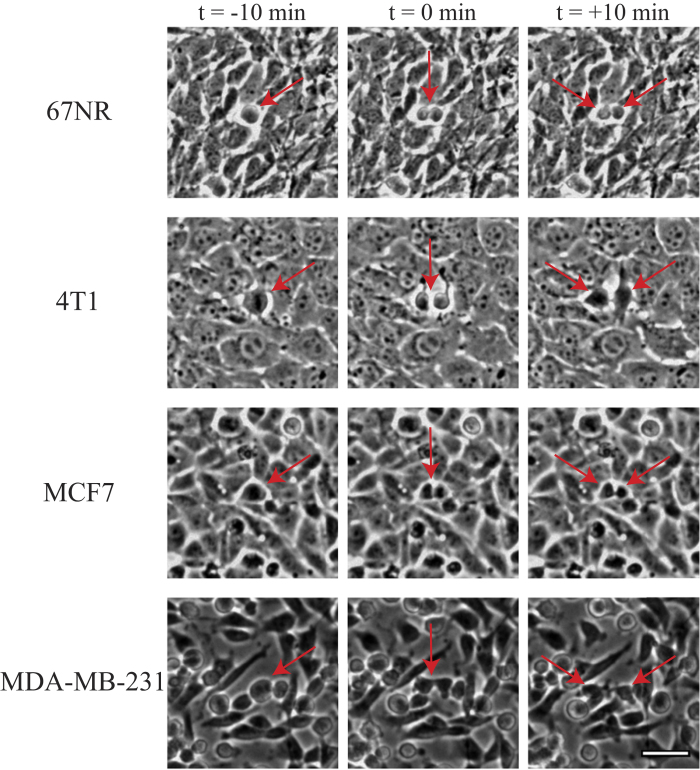
Images of cancer tissue surrounding a cell division site in a confluent monolayer of the breast cancer cell lines 67NR (mouse, non-invasive), 4T1 (mouse, invasive), MCF7 (human, non-invasive), and MDA-MB-231 (human, invasive). The red arrows point to the site of cell division and at the resulting daughter cells. Time zero is defined as the onset of cytokinesis, i.e., the first image where two distinct daughter cells are visible. The dividing cell is centered in the image and the image is rotated so that the daughter cells move in a horizontal direction after cell division. The scalebar is 40 μm and applies to all images.

All tissue types investigated here originate from epithelial monolayer breast tissue and representative images of these monolayers are shown in Fig. 1. We investigated the human breast cancer cell lines MCF7 (non-invasive) and MDA-MB-231 (invasive). These human cell lines show the classical phenotype with the non-invasive MCF7 retaining an epithelial-like and round shape whereas the highly invasive MDA-MB-231 cells exhibit a more mesenchymal-like and elongated appearance (see Fig. 1). In addition, we investigated murine cell lines which exhibit the opposite phenotype with the non-invasive 67NR cells showing a more mesenchymal-like phenotype while the malignant 4T1s maintain a round epithelial shape (see Fig. 1). These differences are also reflected in the gene-expression of the classical epithelial marker E-Cadherin: The invasive human breast cancer cell line MDA-MB-231 shows a striking downregulation of the cell adhesion protein E-Cadherin, while the invasive murine 4T1s maintain high E-Cadherin levels. For the non-invasive cell lines, the human MCF7 exhibit high E-Cadherin levels, while the murine 67NR does not.

The dynamics of the cancerous tissue was quantified by particle image velocimetry (PIV). This method tracks the displacements from image to image by finding the maximum correlation between intensity patterns^13^ (more details are given in Methods). The time lapse between two consecutive images was 2 minutes and the division sites were chosen so that no other divisions took place during this time interval within the frame investigated. An example of an extracted velocity field is shown in Supplementary Fig. S1.

### Correlation between velocity and invasiveness

**Figure 2:**
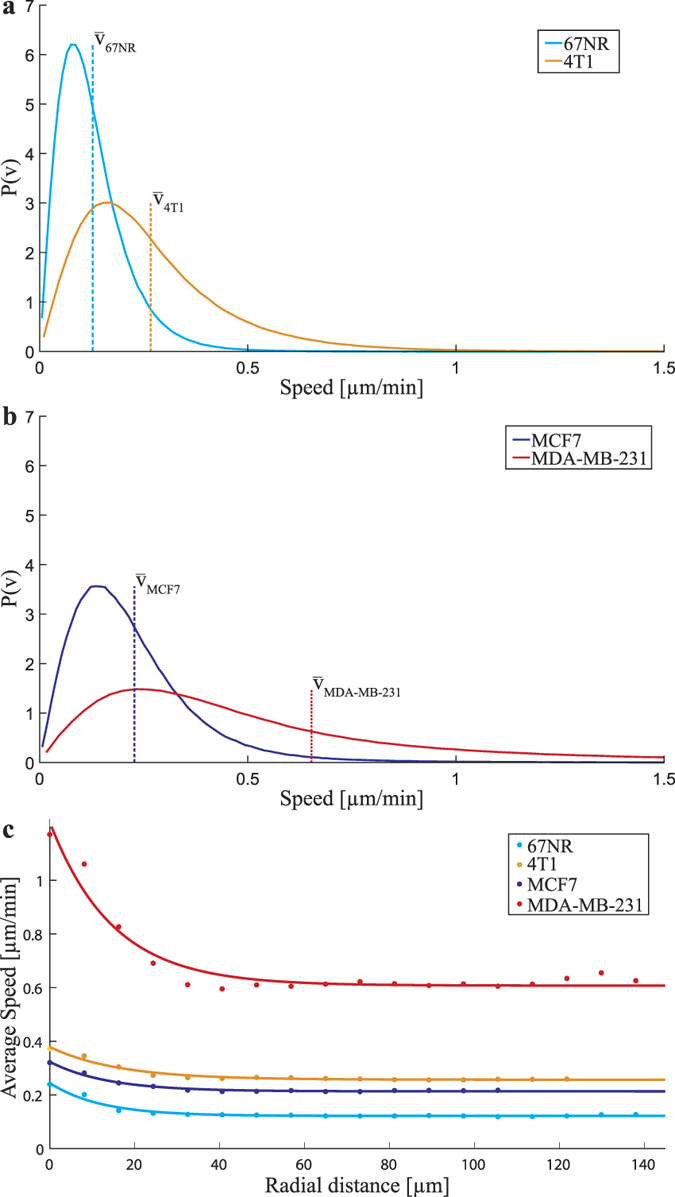
Invasive cancerous tissues move faster than their non-invasive analogue. (**a**) Probability density functions of the speeds from the complete monolayer velocity field (obtained by PIV analysis) during the entire imaging period (80 min) for the human cells (non-invasive MCF7, invasive MDA-MB-231). The vertical lines depict the mean speed for each tissue type. (**b**) Same as (**a**) for the murine cells (non-invasive 67NR, invasive 4T1). (**c**) The average tissue speeds as a function of distance from the division site, dots denote data points, solid lines are exponential fits. The speed is only elevated compared to the normal tissue speeds ~1 cell diameter away from the division site. Also, it is clear from this plot that the most aggressive cell type here investigated, the human MDA-MB-231 cells, move significantly faster than any of the other cell types.

From the velocity fields, we calculated the speed distributions of all investigated cell tissue types. These are depicted in Fig. 2a and b for the murine and human cells, respectively. At least 30 independent data sets were used for each tissue and for each data set images were analyzed 40 minutes before and 40 minutes after cytokinesis, i.e., at least 1200 images were analyzed for each tissue type. Figure 2c shows the average speed as a function of distance from the division site. Only within ~1 cell diameter from the division site does the average speed exceed the typical tissue speed. Also, cell division is rare and changes in the velocity field around the dividing cell are only visible ~4 min before and ~4 min after cytokinesis. Therefore, the average speed distributions (Fig. 2a and b) characterize the motion of the entire cancerous tissue, not of the cell division site per se. The murine cell lines showed an average speed of 0.13 ± 0.03 μm/min for the non-invasive 67NR cells, while the invasive 4T1 cells had an average velocity of 0.27 ± 0.06 μm/min. For the human cell lines, the non-invasive MCF7 cells display a mean velocity of 0.23 ± 0.02 μm/min, while the invasive MDA-MB-231 cells had an average speed of 0.7 ± 0.2 μm/min. For each cell type, the reported error was found as the standard deviation of the mean speeds, calculated for at least 30 independent experiments. Hence, for both the cancerous tissue types, mouse and human, the invasive cells had a significantly higher average speed than the non-invasive counterparts. From Fig. 2a and b it is also apparent that the speed distribution of the most aggressive cells here investigated, the human MDA-MB-231, has a ‘fatter tail’ than the others, this signifies a relatively large number of cells moving extra-ordinarily fast. For the human cell lines, the average velocities here obtained correspond well to those reported in literature in migration assays^14^,^15^,^16^,^17^. For the murine cell lines, there only exists a value for the non-invasive 67NR of 0.03 μm/min^18^, which is somewhat lower than observed here.

### Divergence and vorticity around dividing cancer cells

**Figure 3:**
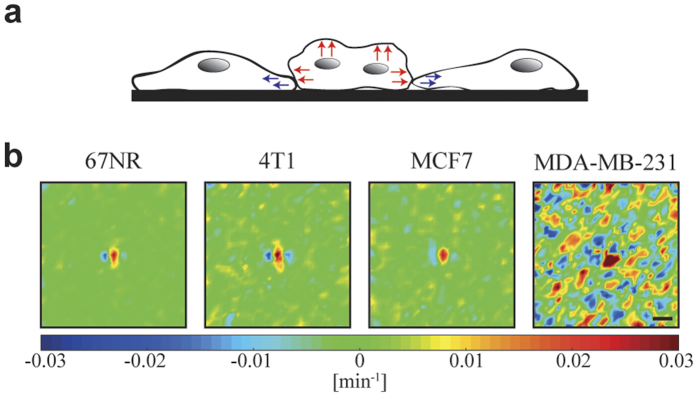
Contraction and expansion in cancer tissue around a cell division site. (**a**) Illustration of the contraction (blue arrows) and expansion (red arrows) of cells undergoing or neighbouring cell division. (**b**) Divergence field during cytokinesis around a dividing cell located in the center of each image. The daughter cells move in a horizontal direction after cytokinesis. Each image is 300 × 300 μm^2^ and is an average of at least 30 data sets. The scalebar is 40 μm and applies to all images. The color scale displays the degree of divergence with blue denoting contraction and red expansion. The two invasive tissue types (mouse 4T1 and human MDA-MB-231) exhibit a higher degree of contraction and expansion than their non-invasive counterparts (mouse 67NR and human MCF7).

**Figure 4:**
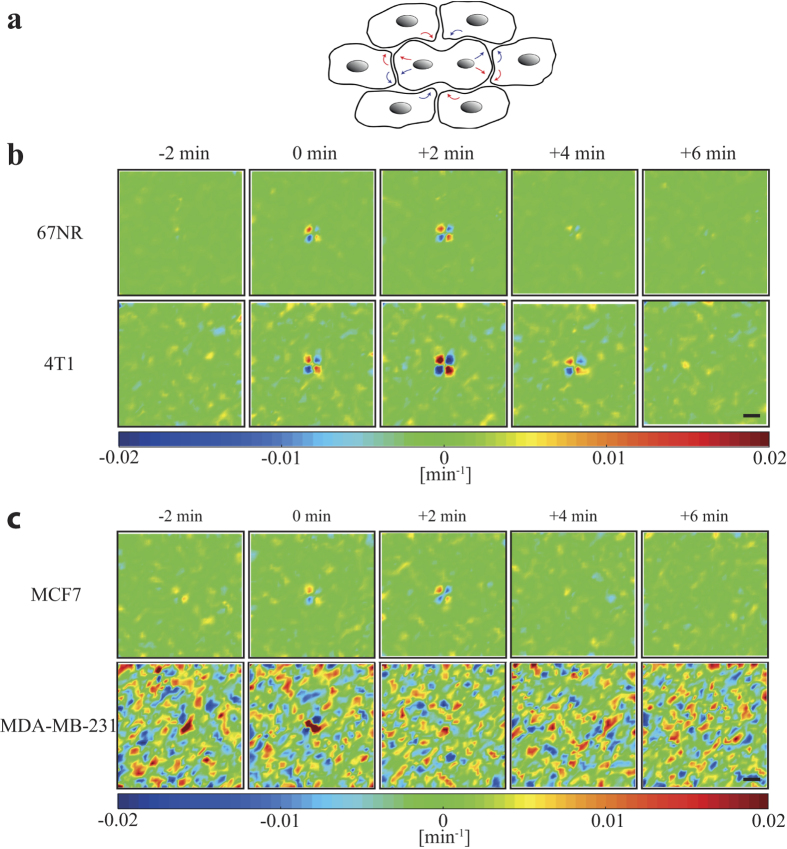
Cell divisions induce ordered vorticity patterns in a confluent monolayer of breast cancer cells. (**a**) Illustration of the division induced cell movement resulting in the emergence of a central vortex pair. (**b**) and (**c**) show the average vorticity fields in an area of 150 μm from a cell division site (n = 30) for breast cancer cells with the daughter cells moving in a horizontal direction after cytokinesis. Time zero was defined as onset of cytokinesis. (**b**) Vorticity fields of murine cell lines (non-invasive 67NR, invasive 4T1). (**c**) Vorticity fields of human cell lines (non-invasive MCF7, invasive MDA-MB-231). The colorscale displays the vorticity (counterclockwise motion blue, and clockwise motion red). The scalebars are 40 μm and apply to all images.

Just before a cancer cell divides it contracts and ‘balls up’ thus becoming higher than the surrounding tissue. At cytokinesis the two daughter cells move in opposite directions away from the cleavage furrow while the adjacent cells contract their cell protrusions (as illustrated in Fig. 3a). These features are visible for all investigated tissue types in the experimentally obtained divergence fields, which are calculated as described in Methods and depicted in Fig. 3b. The invasive cell lines display stronger divergence than the non-invasive counterparts. Also, the human invasive MDA-MB-231 is the tissue type which shows the overall largest degree of divergence throughout the entire tissue (not just around the division site). This is probably because these cells are highly motile and move more independently than other tissue types. To increase the signal to noise ratio, the data shown both in Figs 3b and 4 are averages over at least 30 data sets. Before averaging, the frames were aligned so that the cell division occurs in the center of the image, and rotated so that the two daughter cells move in a horizontal direction immediately following cytokinesis. Similar divergence patterns were previously reported for cell divisions of endothelial cells^6^.

Vorticity is another convenient measure of tissue dynamics, it describes the curl of the velocity field and thereby the swirling induced in the tissue by the dividing cells. After cell division, the two daughter cells expand outward in opposite directions, inducing two pairs of vortices in the tissue, with the center at the division site, see Fig. 4. These two pairs of vortices are visible for at least 4 minutes after cell division in all investigated tissue types. There are, however, important differences between the cell lines: The vorticity around a dividing cell is clearly stronger for the invasive cells (mouse 4T1, human MDA-MB-231) than for their non-invasive counterparts (mouse 67NR, human MCF7). Hence, vorticity correlates with invasiveness. Remarkably, the background level is significantly higher for the most invasive tissue type investigated, the human MDA-MB-231, which was also the case for the divergence (Fig. 3b).

Compared to the vorticity fields surrounding healthy dividing endothelial cells, there are distinct differences to cancer cells. For instance, the vortex pairs induced by cancer cell division are relatively short-lived, they dissipate ~6 minutes after division (Supplementary Fig. S2). In contrast the vortex structures surrounding dividing endothelial cells remain detectable for hours^6^. Also, the extent of the vortex structures around dividing cancer cells is relatively short, only ~1 cell diameter (Supplementary Fig. S2) corresponding to an induction of only primary vortexes. This is quite different from endothelial tissue where the correlation length was significantly larger, expanding over 3 cell diameters and where also secondary and tertiary vortices were induced^6^. Such structured dynamics around dividing cancer cells was not observed in control experiments without cell divisions (Supplementary Fig. S3).

### Continuum model

Biological matter, from single yeast cells^19^ to developing embryos^20^, has been shown to possess viscoelastic properties^21^,^22^. This means that on short time scales, the tissue deforms and relaxes elastically in response to a mechanical loading, whereas loads applied over a longer time will result in an irreversible viscous deformation. Many models of viscoelastic behavior exist and one of the most simple models, which include a crossover from predominantly elastic behavior on short time scales to viscous flow on larger time scales, is the Oldroyd-B model^23^. In the limit of small velocity gradients this model is based on the constitutive relation:


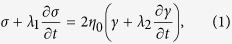


where *σ* is the deviatoric stress tensor, 
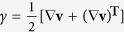
 is the strain rate tensor and the material properties are parametrized by the relaxation time *λ*_1_, the retardation time *λ*_2_ and the total viscosity *η*_0_. In contrast to a purely viscous fluid the stress state of an Oldroyd-B fluid has memory of the past and the constitutive relation therefore includes time derivatives of the stress tensor *σ*. In our model, we go beyond basic viscoelasticity by also including the self-propelling force of individual cells.

The dynamics of the tissue is modeled by the mass and momentum balance equations, where the mass balance is guaranteed by the incompressibility condition ∇ · **v** and the momentum balance equation assumes the form


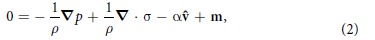


where *ρ* is the mean density, *p* is the pressure, 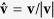 is the direction of the local mean velocity of the tissue, *α* is a positive friction constant and **m** is a stochastic acceleration term describing the self-propelling forces of the cells. The friction term 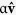 accounts for all the dissipative processes between the cells and the substrate. Similar to the basic Coulomb friction law, we shall here assume that the friction is independent of the speed of the cells. The momentum balance, eq. 2, contains no inertial terms, as the dynamics are assumed to be fully overdamped, i.e., the dissipative forces completely dominate the inertial forces. More details of the model are given under ‘Experimental Procedures’ and in Supplemental Information. The correlation length, *ξ*, in the motion of the cancerous cells is relatively short (Supplementary Table S1 and Supplementary Fig. S4) compared with other tissues such as endothelial tissue^6^, probably because the cancer cells are less tightly connected than the endothelial cells. When tissue dynamics exhibit longer correlation lengths the dynamics are better described by higher order models, see, e.g., in refs 6 and 24.

The governing equations of the model, eqs 1 and 2, are valid in both 2D and 3D. However, the cell-substrate friction term would be irrelevant in 3D, where no substrate is present. Also, one could relatively easily implement the presence of chemokines into the model as a scalar concentration field, the gradient of which would lead to a local force affecting the velocity.

### Comparison to experimental data

The cells are self-propelling and we expect them to explore their environment in a random fashion, when no interactions are taken into account. The motility term **m** in eq. 2 was therefore taken to be the result of an Ornstein-Uhlenbeck process with a noise persistence time *λ*_*m*_, strength *β*_*m*_ and an imposed characteristic length scale 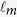 (see Methods). The continuum model described by eqs 1 and 2 was simulated numerically and the speed probability density function *P*(v), the spatial velocity correlation function *C*_*r*_(*r*), and the temporal velocity correlation function *C*_*t*_(*t*) were calculated. The model parameters were fitted by measuring the chi-square between the experimental and the simulated *P*(v), *C*_*r*_(*r*), and *C*_*t*_(*t*), choosing the parameter set yielding the smallest chi-square value in a parameter grid search. (Details on the numerical implementation of the continuum model and the fitted parameters are given in Methods and Supplementary Information).

**Figure 5:**
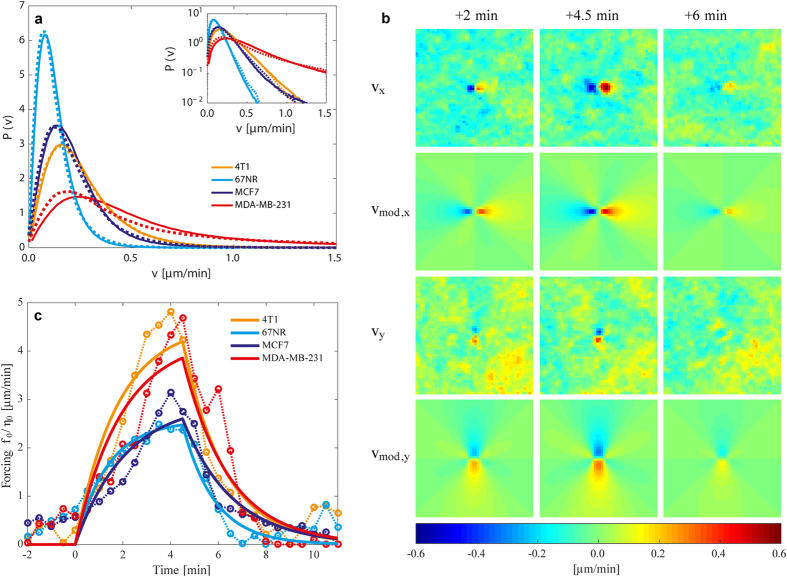
Predictions by the continuum model and comparison to experiments. (**a**) Probability density of the normalized speed distribution during the whole imaging period (80 min). Experimental data are shown by full lines, the model’s predictions by dotted lines. Inset: The speed distributions on a semi-logarithmic scale demonstrating how well the exponential tails are reproduced by the model. (**b**) The velocity field induced by a single cell division ***v*** compares well with a fit of the model velocity field ***v***_***mod***_ given in eq. 4. The cell division occurs at time ***t*** = 0 min and the displayed experimental data ***v***, are from an average over at least 30 data sets. The cell line displayed is the invasive murine 4T1 and the three other cell lines are fitted equally well by the model. (**c**) The force exerted by the expanding daughter cell divided by the viscosity as a function of time. The solid lines are the result of fitting eq. 4 to the experimental velocity time series during division. One value of ***f***_0_/***η***_0_ and ***λ***_2_ is obtained for each of the four time series. The dotted lines represent fits to the same experimental data, when no time dependence is imposed on the model, i.e., a Stokeslet dipole ***v***_***sto***_(***x***) is fitted to each time frame, thus returning one fitted value of ***f***_0_/***η***_0_ per time frame. The time series of ***f***_0_/***η***_0_-values serves as a test of the time dependence predicted by the full time-dependent model. For both the murine and human cells, the invasive cell lines exert the largest force during division and expansion.

Figure 5a shows the speed distributions for the different tissue types returned by the continuum model. It is clear that the model (dotted lines) reproduces the experimental data (full lines) quite well for all tissue types. Even the tails of the speed distributions (see the semi-log inset of Fig. 5b), and the exceptionally ‘fat tail’ of the human invasive MDA-MB-231 in Fig. 2, are captured well by the continuum model, due to the inclusion of viscoelasticity and friction in the model. If these features were not included, the Ornstein-Uhlenbeck forcing **m** would generate a speed distribution with a Gaussian tail. The spatial and temporal correlation functions as well as their fits are shown in Supplementary Fig. S4.

### Quantifying the force exerted by a dividing cell

To quantify the mechanical forces exerted by a dividing cell, we include cell division in the continuum model by adding an active stress *σ*_0_ inspired by refs 25, 26, 27 to the constitutive relation in eq. 2:


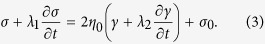


The active stress has a simple on/off time dependence and is assumed to originate from two equal but opposite constant point forces of magnitude *f*_0_ separated by a small distance 2*a* and centered on the cell division site.

We are interested in the cell layer’s response to a division and therefore neglect the noise term **m** in eq. 1 since it represents the cells’ intrinsic motility, which is not a division-induced effect for the studied cell lines. The friction term 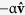 is small compared to the force driving the division and is neglected. In the absence of noise and friction, eqs 1 and 3 can be solved analytically. The obtained velocity field is a Stokeslet dipole **v**_sto_(**x**) in space multiplied by a time dependent function *h*(*t*), (see Methods):


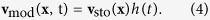


The model velocity field **v**_mod_(**x**, t) was fitted to the time-series of averaged experimental **v** data using regularized Stokeslets^28^. For each time series, the magnitude of the point force divided by the viscosity, *f*_0_/*η*_0_, as well as the retardation time, *λ*_2_, were extracted (values are given in Table 1). A comparison of the experimentally obtained velocities around a dividing cell with the model’s predictions is shown in Fig. 5b. The resulting time dependence of the forcing (defined as force per meter divided by characteristic viscosity) is shown in Fig. 5c and the obtained retardation times, given in Table 1, were similar to the values obtained when fitting the model to the statistical characteristics of the entire velocity field.

As apparent from Fig. 5c and Table 1, the invasive cell lines exerted a larger force to viscosity ratio during cell division than the non-invasive cell lines for both the murine and human model systems. To test the statistical significance of the difference in forcing magnitude *f*_0_/*η*_0_ between invasive and non-invasive cells, a two-sided student’s t-test was performed. A sample of 30 values of *f*_0_/*η*_0_ for each cell type was extracted by fitting the model velocity field **v**_mod_(**x**, t) to each of the 30 individual experimental time series making up the averaged experimental velocity time series **v**. Comparing the samples of 4T1 and 67NR yielded a *p*-value less than 10^−8^ whereas the comparison of MCF7 and MDA-MB-231 yielded a *p*-value less than 10^−3^, supporting that the invasive cell lines 4T1 and MDA-MB-231 do exert a significantly larger forcing *f*_0_/*η*_0_ than their non-invasive analogues. The obtained retardation times, λ_2_, did not differ significantly between cell types, signaling a relative similar rheology.

## Discussion

Using image analysis, we quantified the velocity fields in cancerous tissue surrounding dividing cells. By invoking different measures for the tissue dynamics, speed, divergence and vorticity, we found that invasive cancer cell lines (mouse 4T1 and human MDA-MB-231) move faster, and display stronger divergence and vorticity, than their non-invasive counterparts (mouse 67NR and human MCF7). Hence, fast intrinsic tissue movements correlates with the aggressiveness of the breast cancer type.

The two metastatic cell lines investigated here represent breast cancer cells with a varying level of physical interaction. The MDA-MB-231 are known to have a downregulated production of the classical epithelial marker E-Cadherin^29^, they have a mesenchymal, elongated phenotype, and they primarily migrate and invade as single cells. Although being highly aggressive, 4T1 cells in contrast continue to express the epithelial tight junction marker E-Cadherin^30^ and accordingly migrate in a collective manner^30^,^31^. Despite these striking regulatory differences, our analysis reveals a correlation between invasiveness and all observed parameters characterizing dynamics. In addition, we can exclude a pure correlation between cell shape and dynamics.

All investigated cell lines displayed the emergence of two pairs of vortices around division sites, and the spatial and temporal correlations of these patterns were short compared to similar events in endothelial tissue^6^. This may be attributed to the fact that cancerous tissues have weaker intercellular adhesion than endothelial tissue, and adjacent cells move in a less correlated fashion. Endothelial cells are stress sensitive, tightly packed, and rely on cooperation within the layer to function optimally. Cancer cells, on the other hand, divide at higher rates and their lower cell-to-cell adhesion, compared to normal cells, give rise to high motility.

The continuum model presented here was inspired by the experimental observations, in particular, the relatively short correlation length and the visco-elastic properties of the tissue were crucial for the model’s exact formulation. The model captures the experimentally observed velocity fields, and in addition provides important information on the forces exerted by cancerous cells undergoing division. Our discovery that the invasive cells exert a larger forcing (and a larger force, if viscosity is assumed constant) than the non-invasive counterparts in both the human and murine model system, intuitively makes sense as the more aggressive cells should be better able to squeeze through tight interstitial spaces and even be able to cross boundary layers such as blood vessel walls. The present work focuses on 2D migration and does not consider aspects of tumor growth such as tumor morphology, interaction between healthy and cancerous tissue, nor the availability of resources such as oxygen and nutrients^32^,^33^. The model regards cancer tissue as a continuum and is solely based on the material properties of the system, no biological signaling is included. In real life, the behavior of cancer tissue will be influenced both by biochemical signaling and material properties^34^,^35^,^36^. Although the influence of the mechanics, material properties, and the tumor microenvironment is receiving increasing attention, a full understanding of the mechanisms governing collective dynamics is still missing.

By analyzing the dynamics of cancerous tissue, both murine and human, we found a strong correlation between the invasiveness of breast cancer cell lines and the 2D tissue dynamics. Invasive cell lines, murine 4T1 and human MDA-MB-231, showed significantly faster intrinsic tissue movements than their non-invasive counterparts (murine 67NR and human MCF7). Uncontrolled cell division is a hallmark of cancer cells and the divergence and vorticity fields around dividing cells were significantly stronger for the invasive cell lines than for the non-invasive albeit, with shorter correlation lengths than observed around dividing cells in endothelial tissue^6^. The experimental observations led to formulation of a continuum model which incorporated the viscoelastic nature of the tissue. This model nicely reproduced all observed experimental data, for instance the velocity, divergence and vorticity fields. In addition, the model returned the force^37^ divided by viscosity applied by the dividing cells onto the remainder of the tissue and we found that the forces exerted by the invasive cell lines were significantly larger than by their non-invasive counterparts. These results prove a strong correlation between cancer tissue invasiveness, dynamics and force generation, where the most aggressive cells are the strongest and fastest. This information shows that dynamics are a more reliable parameter for judging aggressiveness than, e.g., cell shape, tissue connectedness, or endothelial marker expression. A natural extension of the current study will be to investigate the dynamics of a tumor embedded in a three dimensional matrix. The theoretical model here presented should still be valid in three dimensions, however, PIV analysis of experimental images would be challenging. *In vivo*, the physical and biological properties of the tumor microenvironment (TME) will influence tumor cell growth^34^ and migration rate^38^. For instance, the rigidity and meshwork density of the TME has been shown to influence cellular migration and invasion^39^,^40^,^41^. Hence, for translation into an *in vivo* or clinical setting, the influence of the TME would need to be assessed.

## Methods

### Cell culture

All cell lines were cultured at 37 °C and with 5% CO_2_. The 4T1 and 67NR murine breast cancer cell lines were a kind gift from Fred Miller (Wayne State University)^37^ and were confirmed through STR testing. The MDA-MB-231 and MCF7 human breast cancer cell lines were purchased from ATCC. All cell lines were routinely tested negative for mycoplasma. The 4T1, 67NR and MDA-MB-231 cells were cultured in Dulbecco’s Modified Eagle Medium (DMEM, Gibco) containing high glucose and GlutaMAX^TM^, 10% Fetal Bovine Serum (FBS) and 1% Penicillin Streptomycin (P/S). The MCF7 cell line was cultured in DMEM/F-12 (Gibco) supplemented with 10% FBS and 1% P/S.

The cells were seeded in Nunc Cell-Culture Treated 6-multiwell plates (Thermoscientific) at a density between 7∙10^5^ to 9∙10^5^ cells. After seeding they were allowed to settle for approximately 24 h (16–32 h) to create a confluent monolayer.

### Time lapse microscopy and image analysis

Phase contrast images were taken of the monolayer for a duration of 6–12 h using a Nikon Eclipse Ti-E microscope system. The majority of the data was taken using a 10x air objective (CFI Plan Fluor DLL, 10x, N.A. 0.30, W.D. 16.0 mm, Ph1, Nikon), acquiring an image every 2 min. To increase spatial and temporal resolution, imaging was repeated with a 20x objective (CFI Plan Fluor DLL, 20x, N.A. 0.50, W.D. 2.1 mm, Ph1, Nikon) taking an image every 0.5 min (see Supplementary Methods for discussion on this).

Dividing cells with a distance of at least 150 μm from other dividing cells during the duration of the observation period were identified manually in the phase contrast images. The dividing cell was centered in the 300 × 300 μm^2^ frame and the frame was rotated so that the daughter cells would move along a horizontal axis away from the site of division (Fig. 1 and Supplementary Fig. S1). The image sequence spanned from +/−40 min from the site of cell division.

### Particle Image Velocimetry, divergence and vorticity

The image processing was performed using particle image velocimetry, more precisely the GUI based open-source tool called PIVlab^13^. This method allows us to perform the analysis of tissue dynamics using Matlab routines. It uses cross correlation algorithms to measure space- and time-resolved flow velocities, and enhances signal-to-noise and vector resolution by applying multiple rounds of displacement analysis to offset the following rounds. These multiple rounds are known as passes. For the PIV analysis, 3 passes were used and final interrogation areas of 15.6 μm for the 10x experiments and 7.8 μm for the 20x experiments, respectively.

From the velocity vectors, it is possible to calculate contraction and expansion of the vector field (divergence), and the swirling tendency (vorticity). These were calculated using the equations below.

Divergence [*min*^−1^]:


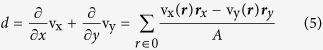


Vorticity [*min*^−1^]:


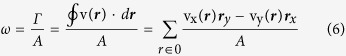


To increase the signal-to-noise ratio, the velocity fields and all subsequent analyses were averaged over at least 30 (for 10x) or 50 (for 20x) data sets, deriving from at least 4 individual experiments. Analysis of the velocities was performed with custom made Matlab scripts.

### The continuum model

The proposed model aims at describing cell-cell interaction, cell-substrate dissipation and intrinsic motility of the cells in a mechanical framework. A continuum description is therefore natural, as it renders mechanical properties such as local stresses and forces easily accessible. The cell-cell interactions are in the continuum described by the material rheology eq. 1, the cell-substrate interactions are accounted for by the friction term 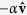 and the intrinsic cell motility is incorporated through the noisy acceleration term **m**.

The acceleration term, representing cell motility, was taken to be the result of an Ornstein-Uhlenbeck process:


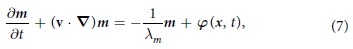


where *λ*_*m*_ is the noise persistence time and ***φ***(***x**, t*) is a white Gaussian noise field ***ζ***(***x**, t*) of strength 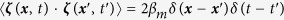, filtered in space with a Gaussian function of width 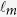 and zero mean to impose a characteristic length scale on the noise. The imposed length scale reflects the fact that a cell is coherent and the velocity fieldhould not fluctuate on scales smaller than the cell size.

### Numerical simulation

The model described by eqs 1 and 2 was simulated numerically in a two-dimensional box with periodic boundaries using a pseudo-spectral method. Non-linear terms were evaluated in real space and then transformed back to Fourier space using the Fast Fourier Transform. An exponential time differencing scheme^42^, was used for the time integration of the stress tensor and the motility term. The velocity field and pressure were found in each time step by a relaxation procedure. A grid of size 256 by 256 and a time step of the order Δ*t~*10^−4^ were used. In physical dimensions this corresponds to a box of length ~200 *μ*m and a time step ~0.01 min.

### The velocity field of a single dividing cell

When friction and motility are ignored, the model described by eqs 1 and 3 can be solved analytically using Laplace transform techniques, since the transformed equations have the sametructure as Newtonian Stokes flow driven by point forces. If the active stress *σ*_0_ is turned on at time *t* = 0 and turned off at time *t* = *t*_*off*_ then the velocity solution **v**_**mod**_(*x, t*) = **v**_**sto**_(*x*)*h*(*t*) has the time dependence:


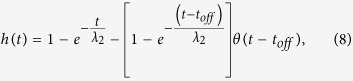


where *θ*(*t*) is the Heaviside step function. We take *σ*_0_ to be the stress resulting from two point forces localted at ***x*** = ±***a*** respectively with equal but opposite force strengths ±***f***_0_. The spatial part of the velocity field is then a sum of the two Stokeslets^28^ corresponding to the two point forces:


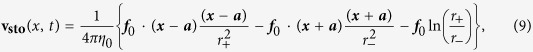


where 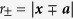 is the distance from the point force located at ***x*** = ±***a***, respectively.

## Additional Information

**How to cite this article:** West, A.-K. V. *et al*. Dynamics of cancerous tissue correlates with invasiveness. *Sci. Rep.* **7**, 43800; doi: 10.1038/srep43800 (2017).

**Publisher's note:** Springer Nature remains neutral with regard to jurisdictional claims in published maps and institutional affiliations.

## Supplementary Material

Supporting Information

## Figures and Tables

**Table 1:** Forcing and typical retardation times for the different cancer model systems, numbers are obtained by fitting the theoretical model to the experimental data.

Cell type	Forcing *f*_0_/*η*_0_	Retardation time *λ*_2_
4T1	(4.6 ± 0.4)*μ*m/min	(1.8 ± 0.3)min
67NR	(2.6 ± 0.4)*μ*m/min	(1.3 ± 0.5)min
MCF7	(2.9 ± 0.4)*μ*m/min	(2.1 ± 0.6)min
MDA-MB-231	(4.3 ± 0.4)*μ*m/min	(1.9 ± 0.3)min
