# Supplementary material for: Dynamics of cancerous tissue correlates with invasiveness
Source: Sci Rep. 2017 Mar 6;7:43800. doi: 10.1038/srep43800 (PMC5338316; doi:10.1038/srep43800)
Supplement: Supporting Information [file srep43800-s1.pdf]

Supplemental Information for Dynamics of cancerous tissue correlates with invasiveness

By Ann-Katrine Vransø West, Lena Wullkopf, Amalie Christensen, Natascha Leijnse,

Jens Magelund Tarp, Joachim Mathiesen, Janine Terra Erler, Lene Broeng Oddershede

Supplemental Figures and Legends

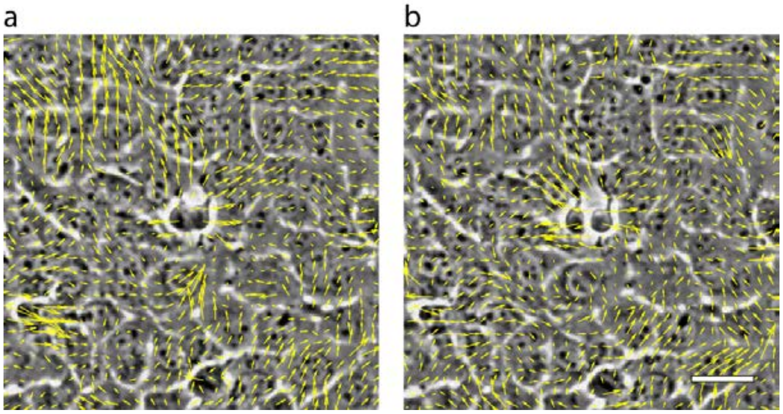

Figure S1. Velocity field around a dividing cell in cancerous 4T1 (mouse, invasive) tissue. A) Image taken at the time defined as zero, onset of cytokinesis. B) Image taken 2 minutes after image A. The monolayer is confluent and the zone between two cells appears bright in these bright field images. The scalebar is 40 μm and applies to both images.

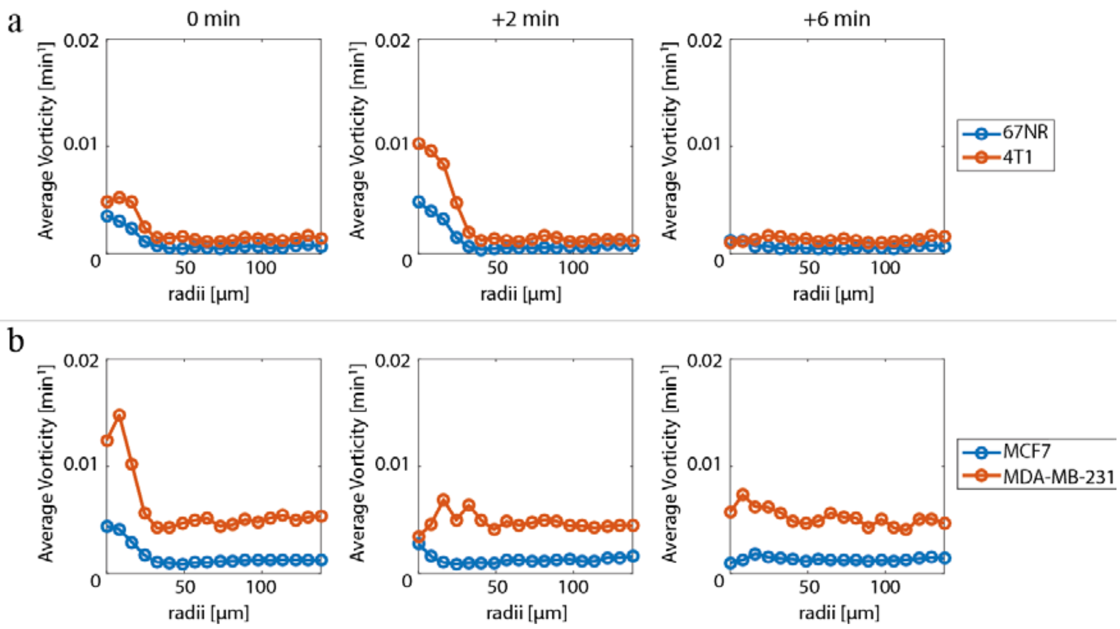

Figure S2. Evolution of vorticity as a function of time where time zero denotes onset of cytokinesis. The upper row is for the mouse tissues, the lower for the human tissues. For both mouse and human tissue the invasive cell lines are plotted with red markers, the non-invasive with blue.

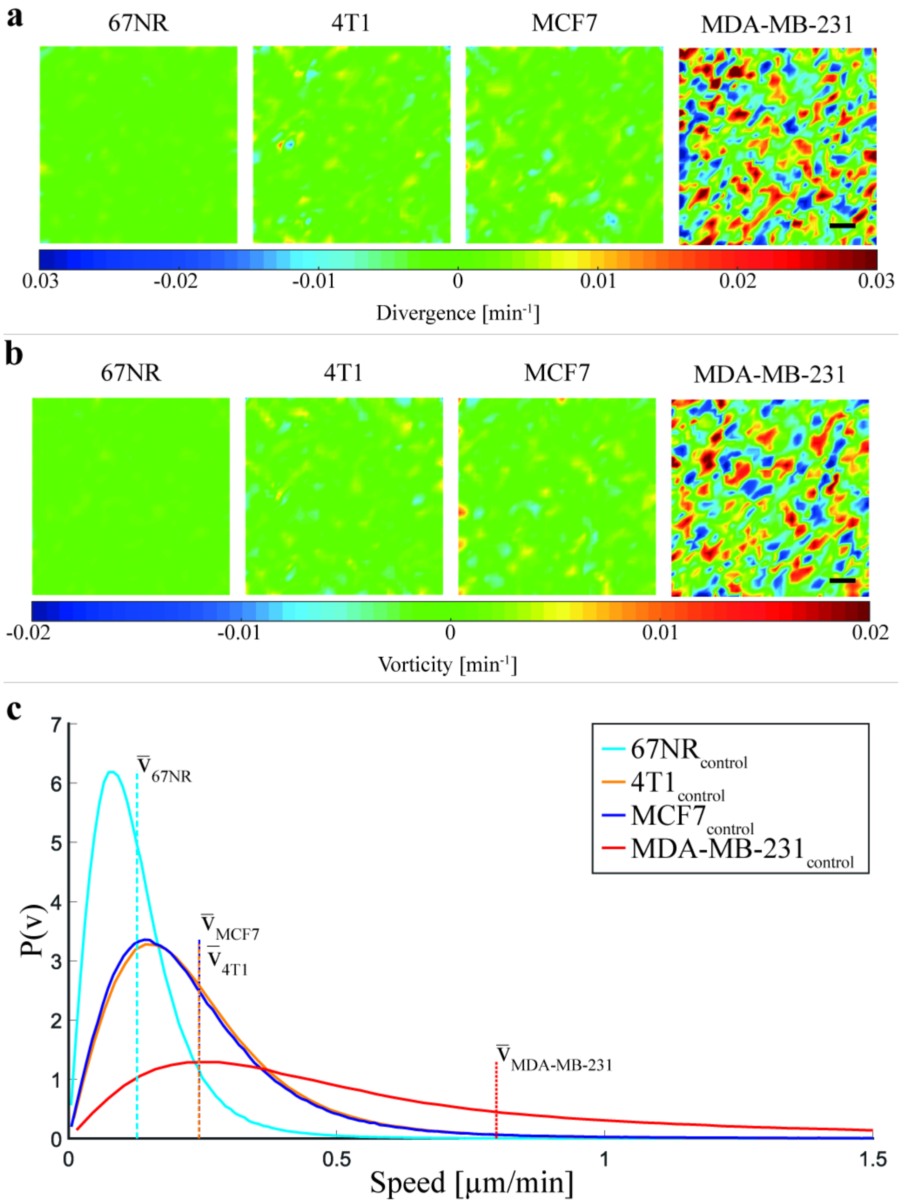

Figure S3. Controls showing the divergence and vortex patterns in random areas without cell divisions. Each image is 300x300 μm, and is averaged over 30 random areas of the cell monolayers. The scalebars are 40 μm and apply to all images. A) Averaged divergence field of random area. B) Averaged vorticity field of random area. C) Probability density distributions of the speed of the random areas in the cell monolayers. Only the most invasive cell line investigated, MDA-MB-231, exhibit significant disturbance and dynamics without cell division. Also, in these controls the speed distributions of the cell monolayers remain unchanged, thus supporting the conclusion that cell division only affects a small area of the total velocity field.

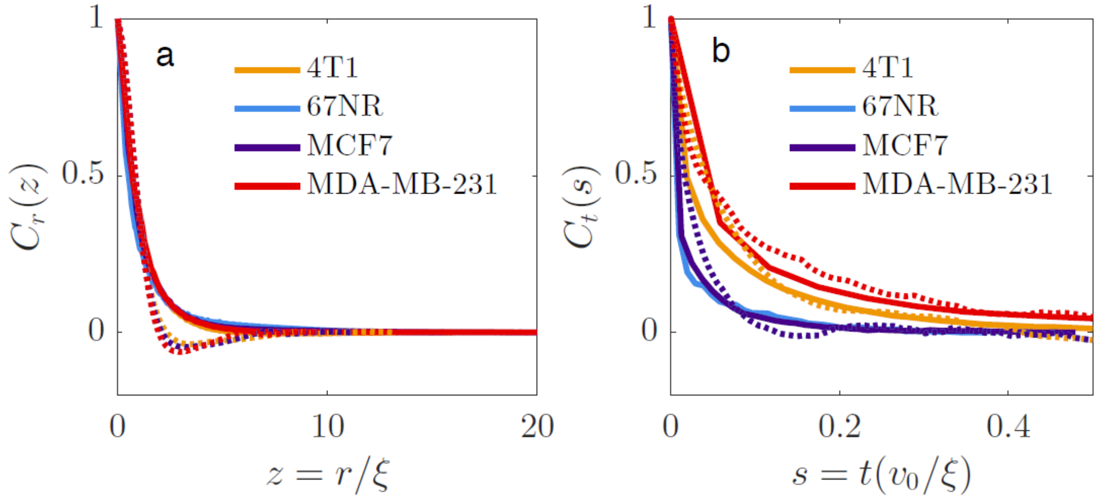

Figure S4. Correlation functions. Full lines represent experimental data, whereas dotted lines are model fits. A) Spatial velocity correlation as a function of the normalized distance $z = r/\xi$, where ξ is the correlation length obtained by fitting $\exp(-r/\xi)$ to the experimental data for each cell type. B) Temporal velocity correlation as a function of the normalized time $s = t\ (v_0/\xi)$, where v_0 is the experimental mean speed. The simulated normalized correlation functions for cell types 67NR and MCF7 are identical and therefore only the MCF7 fit is visible.

Supporting Table

Cell type	λ_1	λ_2	λ_m	ℓ_m	$\beta_m(\rho/\eta_0)^2$	$\alpha(\rho/\eta_0)$
	(min)	(min)	(min)	(µm)	$(\text{min})^{-3}$	$(\mu\text{m} \cdot \text{min})^{-1}$
**4T1**	1.6	0.4	7.8	10	0.003	0.002
**67NR**	2.9	0.7	7.1	12	0.001	0.001
**MCF7**	1.2	0.3	3.0	9	0.010	0.003
**MDA-MB-231**	2.2	0.2	4.3	7	0.125	0.015

Supplementary Table S1. Parameters resulting from fitting the theoretical model to the experimental data.

Supplemental Methods

Choice of microscope objective: The data presented in the main manuscript was acquired by a 10x objective, however, we also acquired a significant amount of data using a 20x objective and with frame sizes of 200x200 □m All conclusions remained the same using these settings. The interrogation area was 15.6 □m (50% step size) for the 10x experiments, while it was 7.8 □m (50% step size) for the 20x experiments. When using the 20x experiments more ‘noisy’ data was picked up as this magnification caught, e.g., the dynamics protrusions and filopodia as well as the Brownian fluctuations of denser intercellular parts (for instance lipid granules).

Model fit and parameters. The six model parameters of Eq. (1) and (2) have the physical dimensions:

$$[\lambda_1, \lambda_2, \lambda_m] = \text{time} \quad [\ell_m] = \text{length} \quad [\beta_m] = \frac{\text{length}^4}{\text{time}^5} \quad [\alpha] = \frac{\text{length}}{\text{time}^2}$$

The density ρ and the viscosity η_0 along with a characteristic speed and length were used to non-dimensionalize the equations. The correlation length was defined as the length scale of the exponentially decaying spatial velocity autocorrelation function $C_r(r) = \exp(-r/\xi)$. Simulation units were converted to physical units by identifying the simulation mean speed v_0 with the experimental mean speed and the simulation correlation length ξ with the experimental correlation length.

The model was fitted to the experimental data by performing a grid search in parameter space and choosing the parameter set which minimized the chi squared between the model and the experimental speed distribution $P(v)$, spatial velocity correlation $C_r(r)$ and temporal $C_t(t)$ correlation, the fitted parameters are given in Supplementary Table S1 below.
